# MicroRNAs as Potential Biomarkers in Merkel Cell Carcinoma

**DOI:** 10.3390/ijms19071873

**Published:** 2018-06-26

**Authors:** Aelita Konstantinell, Dag H. Coucheron, Baldur Sveinbjørnsson, Ugo Moens

**Affiliations:** Molecular Inflammation Research Group, Department of Medical Biology, The Arctic University of Norway, N-9037 Tromsø, Norway; aelita.konstantinell@uit.no (A.K.); dag.coucheron@uit.no (D.H.C.); baldursveinbjornsson@uit.no (B.S.)

**Keywords:** exosomes, extracellular microRNA, large T-antigen, protein-miRNA complex, small t-antigen

## Abstract

Merkel cell carcinoma (MCC) is a rare and aggressive type of skin cancer associated with a poor prognosis. This carcinoma was named after its presumed cell of origin, the Merkel cell, which is a mechanoreceptor cell located in the basal epidermal layer of the skin. Merkel cell polyomavirus seems to be the major causal factor for MCC because approximately 80% of all MCCs are positive for viral DNAs. UV exposure is the predominant etiological factor for virus-negative MCCs. Intracellular microRNA analysis between virus-positive and virus-negative MCC cell lines and tumor samples have identified differentially expressed microRNAs. Comparative microRNA profiling has also been performed between MCCs and other non-MCC tumors, but not between normal Merkel cells and malignant Merkel cells. Finally, Merkel cell polyomavirus encodes one microRNA, but its expression in virus-positive MCCs is low, or non-detectable or absent, jeopardizing its biological relevance in tumorigenesis. Here, we review the results of microRNA studies in MCCs and discuss the potential application of microRNAs as biomarkers for the diagnosis, progression and prognosis, and treatment of MCC.

## 1. MicroRNAs

MicroRNAs (miRNAs) are ~18–24 nucleotides long, non-coding RNA molecules encoded by the genomes of viruses, protists, plants and animals [1]. The human genome may code for more than 3000 miRNAs [2,3]. MiRNAs are produced through multiple processes of larger precursor transcripts referred to as primary miRNAs, which are generated by RNA polymerase II or RNA polymerase III. Primary miRNAs transcribed from genome DNAs are cleaved into precursor miRNAs, which have a short hairpin structure, and subsequently, are exported from the nucleus to the cytoplasm. Lastly, the duplex RNA is processed by degrading one of the strands (the passenger strand) and leaving the other strand as a mature guide miRNA [4,5]. It is also possible to have arm switching, in which the mature guide sequence from a pre-miRNA may shift from one arm to the other in different tissues [6]. In addition to the mature miRNA, isoforms (isomiRs) are also produced that are variants of the mature miRNA. Numerous studies have demonstrated that these isomiRs have functional importance [7,8].

MiRNAs not only reside intracellularly, but also can be released from cells in extracellular vesicles, such as exosomes (vesicles with a characteristic size of ~30–150 nm in diameter), and in apoptotic bodies [9,10,11]. Moreover, extracellular miRNAs in complex with proteins have been described [12]. These circulating miRNAs can be taken up by recipient cells, and in this way, play a role in intercellular communication [13]. It is estimated that approximately 10% of secreted miRNAs are encapsulated in extracellular vesicles, whereas 90% are secreted in a vesicle–free state as complexes with proteins [14]. Plasma from healthy blood donors and media from cell cultures were shown to contain miRNAs associated with the protein argonaute 2 (Ago2), and Ago2-miRNA complexes were stable for at least two months at room temperature. It is not known whether these Ago2-miRNA complexes are byproducts of dying or dead cells or if they are actively released from living cells [12,15]. Recently, neuropilin-1 was identified as a receptor for Ago2-miRNA complexes, suggesting a selective uptake of this protein-miRNA by target cells [16]. Other proteins that have been reported to be associated with extracellular miRNAs are high-density lipoproteins (HDL) and nucleophosmin 1 (NPM1) [17,18,19]. HDL-miRNAs exhibit a distinct expression pattern in relation to different pathological conditions and may thus have biomarker potentials [17,18]. Extracellular miRNAs in complex with NPM1 were detected in a serum-free medium of HepG2 (human hepatocellular), A549 (human lung carcinoma), T98 (human glioblastoma) and BSEA2B (normal human bronchial epithelium) cells [19]. Zernecke and co-workers found that endothelial cell-derived apoptotic bodies generated during atherosclerosis were enriched in miR-126 [20]. Apoptotic bodies have been reported in Merkel cell carcinoma (MCC) [21], but the presence of miRNAs has not been investigated thus far.

Mature miRNAs inhibit gene expression at the posttranscriptional level by binding to complementary sequences in mRNA targets, which prevent their translation or induce their degradation [22]. However, miRNAs can also activate gene expression by binding to target sequences in promoters [23]. MiRNAs can interfere with numerous cellular processes, including cell proliferation, differentiation, development, apoptosis, angiogenesis, metabolism, and immune responses [24,25,26,27,28,29]. An aberrant expression of miRNAs is involved in pathogenic processes, including cancer [30,31,32,33,34,35].

Many human miRNAs are expressed in a cell-type, cellular-process, and disease-specific manner. Moreover, miRNAs are relatively stable. This makes miRNAs relevant as biomarkers for physiological and pathogenic processes. For cancer, in particular, interest in identifying circulating miRNAs as prognostic and diagnostic markers is growing. Both mature miRNA and isomiR profiles may be used as biomarkers [36].

## 2. Merkel Cell Carcinoma

MCC is an aggressive type of cancer as trabecular cell carcinoma of the skin, which was first described by Cyril Toker in 1972 [37]. Later, he showed that the cellular origin of this cancer was Merkel cells; hence, these tumors were renamed MCC. Merkel cells were originally described as Tastzellern or touch cells in the skin by Frederick Sigmund Merkel in 1875 (for a recent review, see [38]), and are located in the basal layer of the skin (in particular, around hair follicles) and mucosa. They serve as mechanoreceptors for gentle touch stimulation, and are associated with afferent sensory nerves to form the Merkel cell-neurite complex. The exact origin of Merkel cells remains controversial. It has been suggest that these cells originate from one of the neurocrest derivatives [39,40,41], keratinocytes, epidermal fibroblasts, early B cells or hair follicle stem cells [42,43,44,45].

MCC is associated with a poor prognosis, as more than one-third of patients die from the disease compared to ~15% for malignant melanoma. Approximately half of MCC patients with advanced diseases survive for nine months or less [46,47]. The highest worldwide incidence of MCCs is found in Australia (1.6 cases per 100,000 persons), followed by Northern America (0.6/100,000) and Europe (~0.3/100,000). The higher incidence in Australia is attributed to high year-round UV exposure [37,47,48,49,50]. The median age at diagnosis is roughly 75 years old, while only 12% of MCC patients are younger than 60 years of age [51]. MCC mostly presents on sun-exposed areas, such as the head and neck and the extremities, and can also occur on the buttocks, oral mucosa, the penis and vulva [52,53,54].

UV light exposure is a major factor for MCC, but immune deficiencies, fair skin, age (immune senescence), association with other cancer, and chronic inflammation can also be contributing factors [47,49,55]. In 2008, a novel virus was identified in eight out of 10 MCC samples [56]. This virus was named Merkel cell polyomavirus (MCPyV), and has subsequently been detected in 80% of all examined MCC samples [57,58]. The oncogenic potentials of this virus are predominantly attributed to two of its viral proteins: large T-antigen (LTAg) and small t-antigen (STAg). Similar to the LTAg and STAg of other polyomaviruses, MCPyV LTAg and STAg can transform cells in vitro and induce tumors in animal models [59,60,61,62,63,64,65,66]. Serological studies of healthy adults showed that ~40–85% of the individuals have antibodies against MCPyV. An age-dependence seroprevalence was measured, increasing from ~10–20% (age 1–5 years) to ~80% (>70 years) [67,68,69,70,71,72,73]. MCPyV seems to be a normal inhabitant of dermal fibroblasts in the skin, and infectious virus particles are chronically shed, thereby suggesting that direct physical contact may be one mode of transmission [74,75]. Whereas the virus is found in an episomal state in non-malignant cells, a characteristic for all virus-positive MCC tumors is that the viral genome is integrated into a clonal pattern, and that a non-sense mutation is present in the LTAg-encoding gene encoding a C-terminal truncated protein. Whether this mutation occurs before or after integration, or whether both scenarios can occur is not known [76,77,78]. The truncated LTAg has lost its ability to support viral replication, but has retained its oncogenic potentials [61,79,80,81].

The MCPyV genome encodes a single miRNA precursor expressed from the late strand, which can produce two mature miRNAs referred to as MCV-miR-M1-5p and MCV-miR-M1-3p [82,83]. MCV-miR-M1-5p seems to be more abundant than MCV-miR-M1-3p in MCPyV-infected neuroectodermal tumor PFSK-1 cells [84], and in HEK293T cells transfected with an expression plasmid encompassing the pre-miR-M1 sequence [85]. The seed sequence is 5′-CUGGAAG-3′ or 5′-GGAAGAA-3′ for MCV-miR1-5p and 5′-UGCUGGA3′- for MCV-miR-M1-3p [82,83,84]. The MCV-miR-M1-5p and MCV-miR-3p sequences are perfectly complementary to the coding sequences of LTAg, hence suggesting that they can repress translation of the LTAg mRNA. Indeed, using a dual-luciferase reporter assay, MCV-miR-M1 was shown to attenuate the expression of LTAg [82,86], whereas Theiss and collaborators showed that this miRNA down-regulates expression of LTAg, limits viral replication, and is necessary to establish a long-term persistent infection of MCPyV-infected neuroectodermal tumor PFSK-1 cells [84]. Based on the seed region, predicted human target genes include genes encoding proteins involved in transcription, cell communication, immune response, apoptosis, autophagy and proteasomal degradation, but it remains to be established if they are genuine targets [82,83,86]. Using HEK293 cells that stably express MCV-miR-M1-5p or MCV-miR-M1-3p, SP100 mRNA was verified as a bona fide target for MCV-miR-M1-5p, but not MCV-miR-M1-3p [86]. This protein is implicated in the innate immune response against dsDNA viruses, including MCPyV [87]. The authors also found that CXCL8 transcript levels were significantly different expressed in stably expressing MCV-miR-M1 cells compared to control cells, but despite the putative MCV-miR-M1-3p seed sequence, this change was indirect and mediated by SP100 [86]. The results of this work suggest that MCPyV uses its miRNA to evade the immune system in order to establish infection, but it also illustrates that the presence of a putative miRNA seed sequence in a target mRNA does not imply that this transcript is targeted by the miRNA. The role of MCV-miR-M1 in MCC tumorigenesis is less clear. Examining the expression of viral miRNA MCV-miR-M1-5p in MCC samples showed that up to 29–80% of the specimens expressed detectable levels of MCV-miR-M1-5p [82,83]. However, the levels of this MCPyV-encoded miRNA in MCCs are low, and are estimated to be less than 0.005% of total miRNA levels [82]. This was confirmed by studies in the MCPyV-positive MCC cell lines WaGa and MKL-1, in which MCV-miR-M1 made up 0.001% of all mature miRNAs [84], and 0.0067%, 0.007%, and 0.0025% of MKL-1a, MKL-1b, MKL-1c cells, respectively [85]. Because its absence or low or undetectable levels, MCV-miR-M1-5p’s biological relevance in cancer and its value as biomarkers are doubtful. The expression of MCV-miR-M1-3p in virus-positive MCCs has not been examined, but as mentioned above, this miRNA is even less abundant than the 5p strand in MCPyV-infected cells.

## 3. Merkel Cell Carcinoma and MicroRNAs

### 3.1. Intracellular MicroRNAs

Ning and colleagues determined the microRNAome by next-generation sequencing of three MCCs, one melanoma, one squamous cell carcinoma (SCC), one basal cell carcinoma (BCC) and one normal skin sample to identify miRNAs specific to MCC [88]. They found that eight miRNAs were upregulated in MCC, while three were downregulated compared to non-MCC cutaneous tumors and normal skin (see Table 1). This differential expression of these miRNAs was confirmed by quantitative reverse transcriptase PCR (qRT-PCR) on a total RNA isolated from 20 MCC samples and from the MCPyV-positive MS-1 MCC cell line. In situ hybridization also confirmed high expression of miR-182 in a MCC sample, but low in surrounding tissue and normal skin. The viral status in the MCC samples was not given. However, the authors also evaluated the expression of four of these MCC- and MS-1-enriched expressed miRNAs (miR-182, miR-183, miR-190b, and miR-340) in the MCPyV-negative cell line MCC13 and found that they demonstrated low expression in these cells. Thus, miR-182, miR-183, miR-190b and miR-340 may be used as biomarkers for MCPyV-positive MCC.

Although tumor-promoting and tumor-inhibiting properties have been attributed to these miRNAs, their biological relevance in MCC remains to be investigated. MiR-182 stimulates metastasis and proliferation, but exerts opposite effects depending on the cancer type [92,93,94,95]. The over-expression of miR-183 inhibits cell migration and invasion in vitro (e.g., [96,97,98]), while other studies demonstrated that miR-183 stimulates cell proliferation and migration [99,100], and is a prognostic biomarker for breast cancer [101]. MiR-190b was shown to inhibit cell proliferation and induce apoptosis in osteosarcoma U2OS cells [102]. MiR-340 has tumor suppressing properties by inhibiting proliferation, invasion and metastasis, and stimulating apoptosis [103,104,105,106,107]. However, miR-340 has also been shown to promote tumor growth in gastric cancer [108]. The miRNAs that had a higher expression in MCPyV-negative MCCs, compared to virus-positive tumors, include miR-125b, miR-374c and miR-3170 [88]. MiR-125b can suppress and promote cancer progression, and further down-regulate γδ T cell activation and cytotoxicity [109], as well as cells involved in anti-tumor surveillance [110]. MiR-374c was identified as a novel miRNA in cervical tumors, and miR-3170 was first identified in the miRNAome of melanoma, which has also been found in breast cancer tumors [111,112]. A possible role of these two miRNAs in cancer has not been elucidated, nor has the plausible involvement in MCC been solved.

A comparison of the microRNAome of MCPyV-positive and MCPyV-negative MCCs revealed that approximately 2.5- to 5-fold higher levels of miR-30a, miR-34a, miR-142-3p and miR-1539 in virus-positive MCCs and 3.5-fold higher levels of miR-181d in virus-negative MCCs [89]. MiR-30a has a dual role in cancer and can act as an oncogene or an onco-suppressor in different cancers, and several of its target genes have been identified (reviewed in [113]). Although miR-34a is a tumor suppressor [114], Veija et al. speculated that an over-expression of miR-34a could decrease p53 expression, thereby interfering with apoptosis, angiogenesis and DNA repair [89]. MiR-142-3p can inhibit cell proliferation and invasion, but high levels have also been correlated with cancer progression [115]. MiR-181d can act as a tumor suppressor [116], but the down-regulation of miR-181d resulted in a decreased proliferation and migration of pancreatic cancer cells [117]. To the best of our knowledge, the role of miR-1539 has not been investigated. The prognostic value of miR-30a, miR-142-3p, miR-1539 and miR-181d is jeopardized, because qRT-PCR validation demonstrated that only miR-34a was significantly under-expressed in virus-negative MCCs compared to virus-positive MCC samples [89]. Whether any of these miRNAs contribute to MCC tumorigenesis remains to be established.

Deep sequencing of RNA purified from normal skin (*n* = 5), BCC (*n* = 5), MCC (*n* = 14), and MCPyV-negative (MCC13, MCC26, UiOS) and MCPyV-positive (MKL-1, MKL-2; MS-1) MCC cells showed that miR-375 is specific for MCC [90]. The miR-375 concentrations were 60-fold higher in the MCC group than in the non-MCC (normal skin and BCC) group. The enrichment of miR-375 seems to be independent of the viral state, because elevated miR-375 levels were found in both virus-negative and virus-positive tumors and tumor cell lines. Of the five skin samples that were examined, three were MCPyV positive, one was virus negative and one was not tested. Of the five BCC samples, four were virus negative and one was not tested. Similarly, since no increased miR-375 levels were found in the virus-positive non-MCC samples, the presence of the virus seems not to affect the expression of miR-375. Although not discussed by the authors, there was a tendency for higher expression levels of miR-9 and miR-188 in MCC samples. MiR-188 suppresses proliferation in different cancers [118,119,120,121]. MiR-9 can stimulate or inhibit cell proliferation and metastasis depending on the type of cancer, whereas high expression levels in most cancers are associated with poor survival of the patients, except for ovarian cancer patients, in which an inverse correlation was found [122,123]. MiR-375 has been described as a tumor suppressor known to impede cell proliferation, to prevent cancer cell migration, and to inhibit autophagy, thereby generating an antitumor effect in liver cancer [124,125,126,127,128,129]. Therefore, it seems surprising that this miRNA is over-expressed in MCC. Nonetheless, an over-expression of miR-375 has also been implied in prostate carcinogenesis and disease progression, while an up-regulation of miR-375 is associated with a poor prognosis in pediatric acute myeloid leukemia [130,131], thus indicating a dual role for miR-375 in cancer. Moreover, miR-375 was shown to inhibit autophagy in hepatocellular carcinoma [132], but whether this role of miR-375 is of importance in MCC is unknown.

A comparison of the intracellular miRNA expression profiles in 10 MCPyV-negative and 16 MCPyV-positive MCCs by a miRNA microarray-based method identified 36 over-expressed and 20 under-expressed miRNAs in virus-positive MCCs compared to virus-negative MCCs [91]. Among these, a significant over-expression of miR-30a-3p, miR-30a-5p, miR-34a, miR-375 and miR-769-5p, and a significant under-expression of miR-203, were confirmed by qRT-PCR. A putative role of miR-30a, miR-34a and miR-375 in oncogenesis was described above. MiR-769 expression was strongly increased in human melanoma cells and clinical tissues compared with their corresponding controls. The over-expression of miR-769 promoted cell proliferation in the human melanoma cell line A375 [133]. MiR-769 may exert these functions by targeting glycogen synthase kinase 3B, while a similar mechanism may be operational in MCC oncogenesis. It is not known whether MCPyV LTAg and/or STAg stimulate the expression of miR-30a-3p, miR-30a-5p, miR-34a, miR-375 and miR-769-5p. The possible involvement of miR-203 in MCC oncogenesis was examined by over-expressing miR-203 in three MCPyV-negative MCC cell lines [91]. This resulted in reduced cell growth, more cells in G1 and less in the G2 phase, but no apparent effect on apoptosis compared to cells transfected with miRNA mimic control. Moreover, survivin expression was reduced. The over-expression of miR-203 in the MCPyV-positive WaGa MCC cell line had no significant effect on cell proliferation, cell cycle progression and survivin expression levels. These results suggest that miR-203 only regulates survivin expression in virus-negative MCCs, but not in MCPyV-positive MCCs, in which LTAg seems to repress survivin expression by sequestering pRb [134]. The same group also examined differentially expressed miRNAs in primary and metastatic MCC tumors [91]. They found that 92 miRNAs were over-expressed in metastasis compared to primary tumors. The four most up-regulated miRNAs were miR-150, miR-142-3p, miR-483-5p and miR-630, but qRT-PCR validation revealed that only miR-150 was significantly overexpressed.

Xie et al. found that miR-375 was specifically over-expressed in MCPyV-positive MCCs, while Renswick et al. reported that miR-375 was specific for MCC, independent of the viral state in the tumors [90,91]. The discrepancy in these results may be explained by the differences in MCC samples that were examined, or because different methods (next-generation sequencing versus miRNA microarray) were used.

The role of MCPyV on miRNA expression in non-small cell lung cancer (NSCLC) was investigated by Lasithiotaki and co-workers [135]. The expression of miR-21, miR-145, miR-146a, miR-155, miR-302c, miR-367 and miR-376c was examined by qRT-PCR in MCPyV-positive and MCPyV-negative NSCLC. MiR-21 and miR-376c were up-regulated, whereas miR-145 was down-regulated in virus-positive NSCLC (*n* = 8) compared to virus-negative NSCLC (*n* = 16). MiR-21 and miR-376c expression levels were also higher in virus-positive NSCLC versus adjacent healthy tissue samples (*n* = 10; 5 MCPyV-positive and 5 MCPyV-negative), while miR-145 levels in MCPyV-negative NSCLC was higher than in control samples. To the best of our knowledge, none of the miRNAs investigated by Lasithiotaki et al. have been described in MCC, except miR-146a which was enriched ~8-fold in exosomes derived from MCPyV-negative MCC13 and MCC26 cell lines compared to virus-positive MKL-1 and MKL-2 cell lines (A.K., D.H.C., B.S., U.M., University of Tromsø, Norway, 2018).

### 3.2. Extracellular MicroRNAs and Merkel Cell Carcinoma

The presence of extracellular miRNA-protein complexes secreted by MCC cell lines or in MCC patients has not been examined thus far. Likewise, the occurrence of miRNAs in apoptotic bodies has not been investigated although apoptotic bodies have been reported in MCC [21]. We have applied next-generation sequencing to examine the microRNAome in exosomes purified from the MCPyV-negative MCC13 and MCC26 and the MCPyV-positive MKL-1 and MKL-2 MCC cell lines. On average, there were 20.4 million reads per sample (three independent exosome samples of each cell line), with the number of miRNAs per sample varying approximately between 200 and 400. Of the previously identified intracellular miRNAs identified in MCC samples of MCC cell lines (Table 1), our preliminary results confirmed the presence of miR-30a, miR-125b, mi-183, miR-190b and miR-375 in exosomes. MCV-miR-M1 was not detected in any of our samples (A.K., D.H.C., B.S., U.M., University of Tromsø, Norway, 2018).

## 4. MicroRNAs as Biomarkers and Therapeutic Targets in Merkel Cell Carcinoma

MiRNAs are key components of cells in both normal and pathogenic states. The miRNA expression pattern of normal cells versus malignant cells differs, and cancer-cell-specific miRNAs are being used as biomarkers in different cancers [136,137,138,139,140,141]. Exosomal miRNAs have become attractive cancer biomarkers because exosomes are easily obtainable from body fluids, such as blood and urine without the requirement of a biopsy sample of the tumor. Exosomal miRNAs in plasma or urine are used as biomarkers for different malignancies, including melanoma, breast, colon, prostate, renal and gastric cancer [142,143,144,145,146,147,148]. Only a few studies have examined the miRNAome of MCC and miRNAs are not yet used as biomarkers. One of the pitfalls of using MCC-derived miRNAs as biomarkers is that the miRNA expression pattern of normal Merkel cells has not been determined because these cells are rare. Hence, it is not known whether the MCC-derived miRNAs are specific for the malignant cells or also expressed by non-malignant Merkel cells. Another problem is the lack of common miRNAs among the MCC expressed miRNAs identified so far by independent studies. MiR-30a, miR-34 and miR-375 were reported by Renwick et al. and Xie et al., but not by others (Table 1) [90,91]. We found miR-30 and miR-375 in exosomes derived from MCC cell lines, but it is not known whether these miRNAs are also present in non-transformed Merkel cells. MiR-30a is expressed in different cell types, including normal dermal fibroblasts, keratinocytes and endothelial cells [149,150,151], whereas miR-375 is present in normal epithelial, pituitary and pancreatic β-cells [152,153,154], jeopardizing the value of these miRNAs as specific biomarkers for MCC. Additional tumor specimens must be investigated in order to isolate MCC-specific miRNAs and their potential use as biomarkers should be verified.

MiRNAs may also be used to determine the viral state in the MCC. Levels of miR-30a and miR-34 were increased in MCPyV-positive MCCs compared to MCPyV-negative [89,91]; hence, these miRNAs may be applied to distinguish between virus-positive and virus-negative cancers. MiR-375 was found to be a specific miRNA for virus-positive MCCs [90,91], but this could not be confirmed by others who found this miRNA in both MCPyV-positive and MCPyV-negative MCC [89] and in exosomes of both virus-positive and virus-negative MCC cell lines (our unpublished results). Whether miR-375 can be used as a hallmark for MCPyV-positive MCCs needs further investigation.

As for diagnostic purposes, real-time PCR methods with primers against exosomal miRNAs specific for MCPyV-positive MCCs could replace the commonly used PCR with MCPyV sequence-specific on DNA extracted from a tumor sample. The advantage of real-time PCR on a circulating miRNA is that body fluids, such as blood or urine, are readily accessible sources and more convenient for the MCC patient to obtain than using a biopsy, and that the number of miRNA molecules can be estimated.

Another criterion for a useful biomarker is that it can predict the outcome of the disease. Studies by Xie et al. found that higher levels of miR-150 were associated with a worse prognosis of MCC [91]. Quantifying MCC-specific miRNA levels may also provide information on the disease progression and the efficiency of treatment in the case of miRNA-target therapy. There is a need for new and improved therapy of MCC patients. As of today, MCC treatment includes surgery, radiotherapy and chemotherapy. In a few cases, a spontaneous regression of primary and metastatic MCC has been reported (see e.g., [155,156,157,158]). Recently, immunotherapy based on blocking the PD1-PDL-1 pathway by either anti-PD1 antibodies (pembrolizumab, nivolumab) or anti-PDL-1 antibodies (avelumab) has demonstrated favorable responses, with a six-month progression-free survival in 40–85%, and even complete resolution of the tumors in some patients [159,160,161,162,163,164,165,166,167]. Avelumab became the first Food and Drug Administration-approved drug for the treatment of MCC (https://www.fda.gov/newsevents/newsroom/pressannouncements/ucm548278.htm, 10 May 2018). Treatment with the anti-CTLA-4 antibody ipilimumab has also shown beneficial effects in metastatic MCC [168,169]. However, not all patients have a positive response, so the development of additional therapies is necessary. Drugs against MCC-specific miRNAs or their target transcripts can supplement immunotherapy. Clinical trials with miRNAs against some pathological conditions except MCC have been initiated [170,171,172]. One of the miRNA-based clinical trials includes miR-34, which is expressed in MCC [89,91].

Finally, miRNAs may also help in solving the enigma of the origin of Merkel cells. MiRNA signatures may be an alternative to immunhistochemical staining. This requires the identification of cell-specific miRNAs of neural crest cells, keratinocytes, epidermal fibroblasts, early B cells and hair follicle stem cells, cells that have been suggested to be the origin of Merkel cells (see Section 2). 

**Figure 1:**
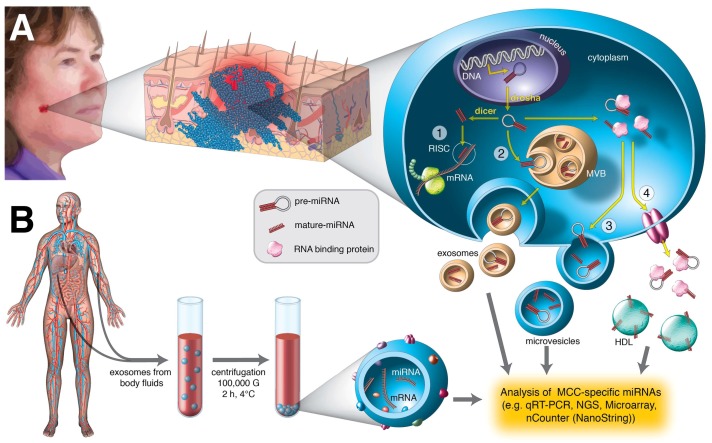
Detection of MCC-specific miRNAs from tumor biopsies or from body fluids. (**A**) The presence of intracellular or/and extracellular MCC-specific miRNAs is examined by qRT-PCR using specific primers. Intracellular miRNAs are amplified from a total RNA isolated from MCC tumor tissue, while extracellular miRNAs are amplified from a RNA extracted from purified exosomes or from the extracellular environment. The biogenesis of a miRNA is shown. A pre-miRNA is transported from the nucleus to the cytoplasm, and when processed to mature miRNA, it binds target mRNA (step 1). Pre-miRNAs and miRNAs can also be enclosed in vesicles and excreted in exosomes (step 2) or other extracellular vesicles (step 3). Pre-miRNAs and miRNAs can also release from the cell in complex with RNA-binding proteins, such as Argonaut 2 or nucleophosmin-1, or in complex with high-density lipoproteins (step 4); (**B**) circulating exosomes are purified from body fluids (e.g., blood, urine, lymphatic fluid, saliva) and a total RNA is extracted. MCC-specific miRNAs are subsequently detected by qRT-PCR applying specific primers, next-generation sequencing (NGS), microarray or nCounter.

In conclusion, multi-center microRNAome studies on a large number of MCC samples or biofluids of patients are required to identify valuable miRNA biomarkers. These data should be linked to parameters, such as the clinical features of the patient, the stage of the tumor (primary or metastatic), viral states and LTAg and STAg expression, age and gender of the patient. MiRNA profiling may be used in determining the prognosis and progression of the disease, and monitoring the response to therapy (Figure 1). Exosomes have been found in a number of biological fluids including plasma, urine, breast milk, semen, cerebrospinal fluid and saliva [173]. Exosomal miRNAs, in addition to intracellular miRNAs, may therefore be easily accessible biomarkers.

## 5. Future Challenges

MiRNAs can be used as reliable biomarkers in several cancers [137,138,139,140,141], and miRNA-based cancer therapy is being developed and tested [170,171]. As outlined above, little research has been done on MCC-specific miRNAs. It is reasonable to wonder whether there is any clinical value for miRNA in MCC and if so, what could it be?

- MCC-specific miRNAs as biomarkers still have a long way to go. Consensus intracellular MCC miRNAs have not yet been identified, and circulating miRNAs have not been investigated. We are currently studying the exosome miRNAome of MCC cell lines with the aim of identifying MCC-specific extracellular miRNAs. Cell–cell communication is important in the tumor microenvironment and one way of communication is exosomes [174]. Thus, analyzing the miRNAome of exosomes may provide clues on how tumor cells promote survival, growth and metastasis by modulating the tumor microenvironment [175].
- The intracellular microRNAome or circulating miRNA may be used to discriminate between MCPyV-negative and MCPyV-positive MCCs. So far, unambiguous miRNAs that allow distinguishing between virus-negative and virus-positive tumors have not yet been described.
- Can MCC-specific miRNAs be used as therapeutic targets? The biological importance of miRNAs in MCC oncogenesis is incompletely understood. In fact, most of the currently reported miRNAs in MCC have dual functions (oncogenic or tumor suppressive roles) in other cell systems, so that targeting their expression may be a double-edged sword. The exact contributing role of miRNAs in MCC is required to design efficient and specific therapies.
- Affordable and easy laboratory tests based on these MCC-specific miRNA biomarkers should be developed to improve the diagnosis, prognosis, and progression of this cancer.

## Figures and Tables

**Table 1:** Differential expressed miRNAs in MCC or MCC-derived cell lines. See the text for details.

Sample (*n*)	Method	miR↑ ^1^	miR↓ ^2^	Reference
MCC (3)	NGS ^3^	miR-7		[88]
		miR-9		
		miR-182		
		miR-183		
		miR-190b		
		miR-340		
		miR-502-3p		
		miR-873		
			miR-125b	
			miR-374c	
			miR-3170	
MS-1	NGS	miR-182		[88]
		miR-183		
		miR-190b		
		miR-340		
MCPyV-positive (15) vs. MCPyV-negative MCC (13)	Microarray	miR-30a		[89]
		miR-34		
		miR-142-3p		
		miR-1539		
			miR-181d	
MCC (14), MKL-1, MKL-2, MS-1 ^4^ versus BCC, normal skin, MCC13, MCC26, UiOS ^5^	NGS	miR-375 ^6^		[90]
MCPyV-positive vs. MCPyV-negative MCC	Microarray	miR-30a-3p		[91]
		miR-30a-5p		
		miR-34a		
		miR-375		
		miR-769-5p		
			miR-203	
Primary vs. metastatic MCC	Microarray	miR-150		[91]

^1^ Higher levels compared to MCPyV-negative MCCs; ^2^ lower levels compared to MCPyV-negative MCCs; ^3^ next-generation sequencing; ^4^ MCPyV-positive MCC cell lines; ^5^ MCPyV-negative MCC cell lines; ^6^ this miR was elevated in MCC tumors and cell lines, independently of the virus state.

## References

[1] He L., Hannon G.J. (2004). MicroRNAs: Small RNAs with a big role in gene regulation. Nat. Rev. Genet..

[2] Kozomara A., Griffiths-Jones S. (2014). miRBase: Annotating high confidence microRNAs using deep sequencing data. Nucleic Acids Res..

[3] Londin E., Loher P., Telonis A.G., Quann K., Clark P., Jing Y., Hatzimichael E., Kirino Y., Honda S., Lally M. (2015). Analysis of 13 cell types reveals evidence for the expression of numerous novel primate- and tissue-specific microRNAs. Proc. Natl. Acad. Sci. USA.

[4] Kim V.N., Han J., Siomi M.C. (2009). Biogenesis of small RNAs in animals. Nat. Rev. Mol. Cell Biol..

[5] Ha M., Kim V.N. (2014). Regulation of microRNA biogenesis. Nat. Rev. Mol. Cell Biol..

[6] Marco A., Ninova M., Griffiths-Jones S. (2013). Multiple products from microRNA transcripts. Biochem. Soc. Trans..

[7] Neilsen C.T., Goodall G.J., Bracken C.P. (2012). IsomiRs—The overlooked repertoire in the dynamic microRNAome. Trends Genet..

[8] Tan G.C., Dibb N. (2015). IsomiRs have functional importance. Malays. J. Pathol..

[9] Lunavat T.R., Cheng L., Kim D.K., Bhadury J., Jang S.C., Lasser C., Sharples R.A., Lopez M.D., Nilsson J., Gho Y.S. (2015). Small RNA deep sequencing discriminates subsets of extracellular vesicles released by melanoma cells—Evidence of unique microRNA cargos. RNA Biol..

[10] Zhang J., Li S., Li L., Li M., Guo C., Yao J., Mi S. (2015). Exosome and exosomal microRNA: Trafficking, sorting, and function. Genom. Proteom. Bioinform..

[11] Turchinovich A., Tonevitsky A.G., Burwinkel B. (2016). Extracellular miRNA: A Collision of Two Paradigms. Trends Biochem. Sci..

[12] Turchinovich A., Weiz L., Langheinz A., Burwinkel B. (2011). Characterization of extracellular circulating microRNA. Nucleic Acids Res..

[13] Hannafon B.N., Ding W.Q. (2013). Intercellular communication by exosome-derived microRNAs in cancer. Int. J. Mol. Sci..

[14] Kai K., Dittmar R.L., Sen S. (2018). Secretory microRNAs as biomarkers of cancer. Semin. Cell Dev. Biol..

[15] Arroyo J.D., Chevillet J.R., Kroh E.M., Ruf I.K., Pritchard C.C., Gibson D.F., Mitchell P.S., Bennett C.F., Pogosova-Agadjanyan E.L., Stirewalt D.L. (2011). Argonaute2 complexes carry a population of circulating microRNAs independent of vesicles in human plasma. Proc. Natl. Acad. Sci. USA.

[16] Prud’homme G.J., Glinka Y., Lichner Z., Yousef G.M. (2016). Neuropilin-1 is a receptor for extracellular miRNA and AGO2/miRNA complexes and mediates the internalization of miRNAs that modulate cell function. Oncotarget.

[17] Vickers K.C., Palmisano B.T., Shoucri B.M., Shamburek R.D., Remaley A.T. (2011). MicroRNAs are transported in plasma and delivered to recipient cells by high-density lipoproteins. Nat. Cell Biol..

[18] Niculescu L.S., Simionescu N., Sanda G.M., Carnuta M.G., Stancu C.S., Popescu A.C., Popescu M.R., Vlad A., Dimulescu D.R., Simionescu M. (2015). MiR-486 and miR-92a Identified in Circulating HDL Discriminate between Stable and Vulnerable Coronary Artery Disease Patients. PLoS ONE.

[19] Wang K., Zhang S., Weber J., Baxter D., Galas D.J. (2010). Export of microRNAs and microRNA-protective protein by mammalian cells. Nucleic Acids Res..

[20] Zernecke A., Bidzhekov K., Noels H., Shagdarsuren E., Gan L., Denecke B., Hristov M., Koppel T., Jahantigh M.N., Lutgens E. (2009). Delivery of microRNA-126 by apoptotic bodies induces CXCL12-dependent vascular protection. Sci. Signal..

[21] Mori Y., Hashimoto K., Tanaka K., Cui C.Y., Mehregan D.R., Stiff M.A. (2001). A study of apoptosis in Merkel cell carcinoma: An immunohistochemical, ultrastructural, DNA ladder, and TUNEL labeling study. Am. J. Dermatopathol..

[22] Iwakawa H.O., Tomari Y. (2015). The Functions of MicroRNAs: MRNA Decay and Translational Repression. Trends Cell Biol..

[23] Place R.F., Li L.C., Pookot D., Noonan E.J., Dahiya R. (2008). MicroRNA-373 induces expression of genes with complementary promoter sequences. Proc. Natl. Acad. Sci. USA.

[24] Wilfred B.R., Wang W.X., Nelson P.T. (2008). Energizing miRNA research: A review of the role of miRNAs in lipid metabolism, with a prediction that miR-103/107 regulates human metabolic pathways. Proc. Natl. Acad. Sci. USA.

[25] Stefani G., Slack F.J. (2008). Small non-coding RNAs in animal development. Nat. Rev. Mol. Cell Biol..

[26] Shi Y., Jin Y. (2009). MicroRNA in cell differentiation and development. Sci. China C Life Sci..

[27] O’Connell R.M., Rao D.S., Chaudhuri A.A., Baltimore D. (2010). Physiological and pathological roles for microRNAs in the immune system. Nat. Rev. Immunol..

[28] Subramanian S., Steer C.J. (2010). MicroRNAs as gatekeepers of apoptosis. J. Cell. Physiol..

[29] Landskroner-Eiger S., Moneke I., Sessa W.C. (2013). miRNAs as modulators of angiogenesis. Cold Spring Harb. Perspect. Med..

[30] Zhao Y., Srivastava D. (2007). A developmental view of microRNA function. Trends Biochem. Sci..

[31] Visone R., Croce C.M. (2009). MiRNAs and cancer. Am. J. Pathol..

[32] Ameres S.L., Zamore P.D. (2013). Diversifying microRNA sequence and function. Nat. Rev. Mol. Cell Biol..

[33] Vidigal J.A., Ventura A. (2015). The biological functions of miRNAs: Lessons from in vivo studies. Trends Cell Biol..

[34] Bracken C.P., Scott H.S., Goodall G.J. (2016). A network-biology perspective of microRNA function and dysfunction in cancer. Nat. Rev. Genet..

[35] Lou W., Liu J., Gao Y., Zhong G., Chen D., Shen J., Bao C., Xu L., Pan J., Cheng J. (2017). MicroRNAs in cancer metastasis and angiogenesis. Oncotarget.

[36] Telonis A.G., Magee R., Loher P., Chervoneva I., Londin E., Rigoutsos I. (2017). Knowledge about the presence or absence of miRNA isoforms (isomiRs) can successfully discriminate amongst 32 TCGA cancer types. Nucleic Acids Res..

[37] Schrama D., Ugurel S., Becker J.C. (2012). Merkel cell carcinoma: Recent insights and new treatment options. Curr. Opin. Oncol..

[38] Erovic I., Erovic B.M. (2013). Merkel cell carcinoma: The past, the present, and the future. J. Skin Cancer.

[39] Godlewski J., Kowalczyk A., Kozielec Z., Pidsudko Z., Kmiec A., Siedlecka-Kroplewska K. (2013). Plasticity of neuropeptidergic neoplasm cells in the primary and metastatic Merkel cell carcinoma. Folia Histochem. Cytobiol..

[40] Visscher D., Cooper P.H., Zarbo R.J., Crissman J.D. (1989). Cutaneous neuroendocrine (Merkel cell) carcinoma: An immunophenotypic, clinicopathologic, and flow cytometric study. Mod. Pathol..

[41] Love J.E., Thompson K., Kilgore M.R., Westerhoff M., Murphy C.E., Papanicolau-Sengos A., McCormick K.A., Shankaran V., Vandeven N., Miller F. (2017). CD200 Expression in Neuroendocrine Neoplasms. Am. J. Clin. Pathol..

[42] Jankowski M., Kopinski P., Schwartz R., Czajkowski R. (2014). Merkel cell carcinoma: Is this a true carcinoma?. Exp. Dermatol..

[43] Sauer C.M., Haugg A.M., Chteinberg E., Rennspiess D., Winnepenninckx V., Speel E.J., Becker J.C., Kurz A.K., Zur Hausen A. (2017). Reviewing the current evidence supporting early B-cells as the cellular origin of Merkel cell carcinoma. Crit. Rev. Oncol. Hematol..

[44] Tilling T., Moll I. (2012). Which are the cells of origin in merkel cell carcinoma?. J. Skin Cancer.

[45] Sunshine J.C., Jahchan N.S., Sage J., Choi J. (2018). Are there multiple cells of origin of Merkel cell carcinoma?. Oncogene.

[46] Tai P. (2008). Merkel cell cancer: Update on biology and treatment. Curr. Opin. Oncol..

[47] Becker J.C., Stang A., DeCaprio J.A., Cerroni L., Lebbe C., Veness M., Nghiem P. (2017). Merkel cell carcinoma. Nat. Rev. Dis. Primers.

[48] Agelli M., Clegg L.X., Becker J.C., Rollison D.E. (2010). The etiology and epidemiology of merkel cell carcinoma. Curr. Probl. Cancer.

[49] Kaae J., Hansen A.V., Biggar R.J., Boyd H.A., Moore P.S., Wohlfahrt J., Melbye M. (2010). Merkel cell carcinoma: Incidence, mortality, and risk of other cancers. J. Natl. Cancer Inst..

[50] Stang A., Becker J.C., Nghiem P., Ferlay J. (2018). The association between geographic location and incidence of Merkel cell carcinoma in comparison to melanoma: An international assessment. Eur. J. Cancer.

[51] Harms K.L., Healy M.A., Nghiem P., Sober A.J., Johnson T.M., Bichakjian C.K., Wong S.L. (2016). Analysis of Prognostic Factors from 9387 Merkel Cell Carcinoma Cases Forms the Basis for the New 8th Edition AJCC Staging System. Ann. Surg. Oncol..

[52] Tomic S., Warner T.F., Messing E., Wilding G. (1995). Penile Merkel cell carcinoma. Urology.

[53] Roy S., Das I., Nandi A., Roy R. (2015). Primary Merkel cell carcinoma of the oral mucosa in a young adult male: Report of a rare case. Indian J. Pathol. Microbiol..

[54] Nguyen A.H., Tahseen A.I., Vaudreuil A.M., Caponetti G.C., Huerter C.J. (2017). Clinical features and treatment of vulvar Merkel cell carcinoma: A systematic review. Gynecol. Oncol. Res. Pract..

[55] Oram C.W., Bartus C.L., Purcell S.M. (2016). Merkel cell carcinoma: A review. Cutis.

[56] Feng H., Shuda M., Chang Y., Moore P.S. (2008). Clonal integration of a polyomavirus in human Merkel cell carcinoma. Science.

[57] Coursaget P., Samimi M., Nicol J.T., Gardair C., Touze A. (2013). Human Merkel cell polyomavirus: Virological background and clinical implications. APMIS.

[58] Liu W., MacDonald M., You J. (2016). Merkel cell polyomavirus infection and Merkel cell carcinoma. Curr. Opin. Virol..

[59] Baez C.F., Brandao Varella R., Villani S., Delbue S. (2017). Human Polyomaviruses: The Battle of Large and Small Tumor Antigens. Virology.

[60] Wendzicki J.A., Moore P.S., Chang Y. (2015). Large T and small T antigens of Merkel cell polyomavirus. Curr. Opin. Virol..

[61] Demetriou S.K., Ona-Vu K., Sullivan E.M., Dong T.K., Hsu S.W., Oh D.H. (2012). Defective DNA repair and cell cycle arrest in cells expressing Merkel cell polyomavirus T antigen. Int. J. Cancer.

[62] Cheng J., Rozenblatt-Rosen O., Paulson K.G., Nghiem P., DeCaprio J.A. (2013). Merkel cell polyomavirus large T antigen has growth-promoting and inhibitory activities. J. Virol..

[63] Verhaegen M.E., Mangelberger D., Harms P.W., Vozheiko T.D., Weick J.W., Wilbert D.M., Saunders T.L., Ermilov A.N., Bichakjian C.K., Johnson T.M. (2015). Merkel cell polyomavirus small T antigen is oncogenic in transgenic mice. J. Investig. Dermatol..

[64] Spurgeon M.E., Cheng J., Bronson R.T., Lambert P.F., DeCaprio J.A. (2015). Tumorigenic activity of merkel cell polyomavirus T antigens expressed in the stratified epithelium of mice. Cancer Res..

[65] Shuda M., Guastafierro A., Geng X., Shuda Y., Ostrowski S.M., Lukianov S., Jenkins F.J., Honda K., Maricich S.M., Moore P.S. (2015). Merkel Cell Polyomavirus Small T Antigen Induces Cancer and Embryonic Merkel Cell Proliferation in a Transgenic Mouse Model. PLoS ONE.

[66] Verhaegen M.E., Mangelberger D., Harms P.W., Eberl M., Wilbert D.M., Meireles J., Bichakjian C.K., Saunders T.L., Wong S.Y., Dlugosz A.A. (2017). Merkel Cell Polyomavirus Small T Antigen Initiates Merkel Cell Carcinoma-like Tumor Development in Mice. Cancer Res..

[67] Kean J.M., Rao S., Wang M., Garcea R.L. (2009). Seroepidemiology of human polyomaviruses. PLoS Pathog..

[68] Tolstov Y.L., Pastrana D.V., Feng H., Becker J.C., Jenkins F.J., Moschos S., Chang Y., Buck C.B., Moore P.S. (2009). Human Merkel cell polyomavirus infection II. MCV is a common human infection that can be detected by conformational capsid epitope immunoassays. Int. J. Cancer.

[69] Pastrana D.V., Tolstov Y.L., Becker J.C., Moore P.S., Chang Y., Buck C.B. (2009). Quantitation of human seroresponsiveness to Merkel cell polyomavirus. PLoS Pathog..

[70] Viscidi R.P., Rollison D.E., Sondak V.K., Silver B., Messina J.L., Giuliano A.R., Fulp W., Ajidahun A., Rivanera D. (2011). Age-specific seroprevalence of Merkel cell polyomavirus, BK virus, and JC virus. Clin. Vaccine Immunol..

[71] Nicol J.T., Robinot R., Carpentier A., Carandina G., Mazzoni E., Tognon M., Touze A., Coursaget P. (2013). Age-specific seroprevalences of merkel cell polyomavirus, human polyomaviruses 6, 7, and 9, and trichodysplasia spinulosa-associated polyomavirus. Clin. Vaccine Immunol..

[72] Zhang C., Liu F., He Z., Deng Q., Pan Y., Liu Y., Zhang C., Ning T., Guo C., Liang Y. (2014). Seroprevalence of Merkel cell polyomavirus in the general rural population of Anyang, China. PLoS ONE.

[73] Antonsson A., Neale R.E., O’Rourke P., Wockner L., Michel A., Pawlita M., Waterboer T., Green A.C. (2018). Prevalence and stability of antibodies to thirteen polyomaviruses and association with cutaneous squamous cell carcinoma: A population-based study. Clin. Virol..

[74] Schowalter R.M., Pastrana D.V., Pumphrey K.A., Moyer A.L., Buck C.B. (2010). Merkel cell polyomavirus and two previously unknown polyomaviruses are chronically shed from human skin. Cell Host Microbe.

[75] Martel-Jantin C., Pedergnana V., Nicol J.T., Leblond V., Tregouet D.A., Tortevoye P., Plancoulaine S., Coursaget P., Touze A., Abel L. (2013). Merkel cell polyomavirus infection occurs during early childhood and is transmitted between siblings. J. Clin. Virol..

[76] Stakaityte G., Wood J.J., Knight L.M., Abdul-Sada H., Adzahar N.S., Nwogu N., Macdonald A., Whitehouse A. (2014). Merkel cell polyomavirus: Molecular insights into the most recently discovered human tumour virus. Cancers.

[77] Arora R., Chang Y., Moore P.S. (2012). MCV and Merkel cell carcinoma: A molecular success story. Curr. Opin. Virol..

[78] Chang Y., Moore P.S. (2012). Merkel cell carcinoma: A virus-induced human cancer. Annu. Rev. Pathol..

[79] Houben R., Adam C., Baeurle A., Hesbacher S., Grimm J., Angermeyer S., Henzel K., Hauser S., Elling R., Brocker E.B. (2012). An intact retinoblastoma protein-binding site in Merkel cell polyomavirus large T antigen is required for promoting growth of Merkel cell carcinoma cells. Int. J. Cancer.

[80] Hesbacher S., Pfitzer L., Wiedorfer K., Angermeyer S., Borst A., Haferkamp S., Scholz C.J., Wobser M., Schrama D., Houben R. (2016). RB1 is the crucial target of the Merkel cell polyomavirus Large T antigen in Merkel cell carcinoma cells. Oncotarget.

[81] Shuda M., Kwun H.J., Feng H., Chang Y., Moore P.S. (2011). Human Merkel cell polyomavirus small T antigen is an oncoprotein targeting the 4E-BP1 translation regulator. J. Clin. Investig..

[82] Seo G.J., Chen C.J., Sullivan C.S. (2009). Merkel cell polyomavirus encodes a microRNA with the ability to autoregulate viral gene expression. Virology.

[83] Lee S., Paulson K.G., Murchison E.P., Afanasiev O.K., Alkan C., Leonard J.H., Byrd D.R., Hannon G.J., Nghiem P. (2011). Identification and validation of a novel mature microRNA encoded by the Merkel cell polyomavirus in human Merkel cell carcinomas. J. Clin. Virol..

[84] Theiss J.M., Gunther T., Alawi M., Neumann F., Tessmer U., Fischer N., Grundhoff A. (2015). A Comprehensive Analysis of Replicating Merkel Cell Polyomavirus Genomes Delineates the Viral Transcription Program and Suggests a Role for mcv-miR-M1 in Episomal Persistence. PLoS Pathog..

[85] Chen C.J., Cox J.E., Azarm K.D., Wylie K.N., Woolard K.D., Pesavento P.A., Sullivan C.S. (2015). Identification of a polyomavirus microRNA highly expressed in tumors. Virology.

[86] Akhbari P., Tobin D., Poterlowicz K., Roberts W., Boyne J.R. (2018). MCV-miR-M1 targets the host-cell immune response resulting in the attenuation of neutrophil chemotaxis. J. Investig. Dermatol..

[87] Neumann F., Czech-Sioli M., Dobner T., Grundhoff A., Schreiner S., Fischer N. (2016). Replication of Merkel cell polyomavirus induces reorganization of promyelocytic leukemia nuclear bodies. J. Gen. Virol..

[88] Ning M.S., Kim A.S., Prasad N., Levy S.E., Zhang H., Andl T. (2014). Characterization of the Merkel Cell Carcinoma miRNome. J. Skin Cancer.

[89] Veija T., Sahi H., Koljonen V., Bohling T., Knuutila S., Mosakhani N. (2015). miRNA-34a underexpressed in Merkel cell polyomavirus-negative Merkel cell carcinoma. Virchows Arch..

[90] Renwick N., Cekan P., Masry P.A., McGeary S.E., Miller J.B., Hafner M., Li Z., Mihailovic A., Morozov P., Brown M. (2013). Multicolor microRNA FISH effectively differentiates tumor types. J. Clin. Investig..

[91] Xie H., Lee L., Caramuta S., Hoog A., Browaldh N., Bjornhagen V., Larsson C., Lui W.O. (2014). MicroRNA expression patterns related to merkel cell polyomavirus infection in human merkel cell carcinoma. J. Investig. Dermatol..

[92] Segura M.F., Hanniford D., Menendez S., Reavie L., Zou X., Alvarez-Diaz S., Zakrzewski J., Blochin E., Rose A., Bogunovic D. (2009). Aberrant miR-182 expression promotes melanoma metastasis by repressing FOXO3 and microphthalmia-associated transcription factor. Proc. Natl. Acad. Sci. USA.

[93] Tang L., Chen F., Pang E.J., Zhang Z.Q., Jin B.W., Dong W.F. (2015). MicroRNA-182 inhibits proliferation through targeting oncogenic ANUBL1 in gastric cancer. Oncol. Rep..

[94] Feng Y.A., Liu T.E., Wu Y. (2015). microRNA-182 inhibits the proliferation and migration of glioma cells through the induction of neuritin expression. Oncol. Lett..

[95] Zhang X., Ma G., Liu J., Zhang Y. (2017). MicroRNA-182 promotes proliferation and metastasis by targeting FOXF2 in triple-negative breast cancer. Oncol. Lett..

[96] Lowery A.J., Miller N., Dwyer R.M., Kerin M.J. (2010). Dysregulated miR-183 inhibits migration in breast cancer cells. BMC Cancer.

[97] Miao F., Zhu J., Chen Y., Tang N., Wang X., Li X. (2016). MicroRNA-183-5p promotes the proliferation, invasion and metastasis of human pancreatic adenocarcinoma cells. Oncol. Lett..

[98] Yang X., Wang L., Wang Q., Li L., Fu Y., Sun J. (2018). MiR-183 inhibits osteosarcoma cell growth and invasion by regulating LRP6-Wnt/beta-catenin signaling pathway. Biochem. Biophys. Res. Commun..

[99] Ren L.H., Chen W.X., Li S., He X.Y., Zhang Z.M., Li M., Cao R.S., Hao B., Zhang H.J., Qiu H.Q. (2014). MicroRNA-183 promotes proliferation and invasion in oesophageal squamous cell carcinoma by targeting programmed cell death. Br. J. Cancer.

[100] Ruan H., Liang X., Zhao W., Ma L., Zhao Y. (2017). The effects of microRNA-183 promots cell proliferation and invasion by targeting MMP-9 in endometrial cancer. Biomed. Pharmacother..

[101] Song C., Zhang L., Wang J., Huang Z., Li X., Wu M., Li S., Tang H., Xie X. (2016). High expression of microRNA-183/182/96 cluster as a prognostic biomarker for breast cancer. Sci. Rep..

[102] Kang M., Xia P., Hou T., Qi Z., Liao S., Yang X. (2017). MicroRNA-190b inhibits tumor cell proliferation and induces apoptosis by regulating Bcl-2 in U2OS osteosarcoma cells. Die Pharm..

[103] Huang K., Tang Y., He L., Dai Y. (2016). MicroRNA-340 inhibits prostate cancer cell proliferation and metastasis by targeting the MDM2-p53 pathway. Oncol. Rep..

[104] Yuan J., Ji H., Xiao F., Lin Z., Zhao X., Wang Z., Zhao J., Lu J. (2017). MicroRNA-340 inhibits the proliferation and invasion of hepatocellular carcinoma cells by targeting JAK1. Biochem. Biophys. Res. Commun..

[105] Maskey N., Li D., Xu H., Song H., Wu C., Hua K., Song J., Fang L. (2017). MicroRNA-340 inhibits invasion and metastasis by downregulating ROCK1 in breast cancer cells. Oncol. Lett..

[106] Arivazhagan R., Lee J., Bayarsaikhan D., Kwak P., Son M., Byun K., Salekdeh G.H., Lee B. (2018). MicroRNA-340 inhibits the proliferation and promotes the apoptosis of colon cancer cells by modulating REV3L. Oncotarget.

[107] Qu F., Wang X. (2017). microRNA-340 induces apoptosis by downregulation of BAG3 in ovarian cancer SKOV3 cells. Pharmazie.

[108] Yin G., Zhou H., Xue Y., Yao B., Zhao W. (2016). MicroRNA-340 promotes the tumor growth of human gastric cancer by inhibiting cyclin G2. Oncol. Rep..

[109] Yin H., Sun Y., Wang X., Park J., Zhang Y., Li M., Yin J., Liu Q., Wei M. (2015). Progress on the relationship between miR-125 family and tumorigenesis. Exp. Cell Res..

[110] Zou C., Zhao P., Xiao Z., Han X., Fu F., Fu L. (2017). Gammadelta T cells in cancer immunotherapy. Oncotarget.

[111] Stark M.S., Tyagi S., Nancarrow D.J., Boyle G.M., Cook A.L., Whiteman D.C., Parsons P.G., Schmidt C., Sturm R.A., Hayward N.K. (2010). Characterization of the Melanoma miRNAome by Deep Sequencing. PLoS ONE.

[112] Persson H., Kvist A., Rego N., Staaf J., Vallon-Christersson J., Luts L., Loman N., Jonsson G., Naya H., Hoglund M. (2011). Identification of new microRNAs in paired normal and tumor breast tissue suggests a dual role for the ERBB2/Her2 gene. Cancer Res..

[113] Yang X., Chen Y., Chen L. (2017). The Versatile Role of microRNA-30a in Human Cancer. Cell. Physiol. Biochem..

[114] Farooqi A.A., Tabassum S., Ahmad A. (2017). MicroRNA-34a: A Versatile Regulator of Myriads of Targets in Different Cancers. Int. J. Mol. Sci..

[115] Shrestha A., Mukhametshina R.T., Taghizadeh S., Vasquez-Pacheco E., Cabrera-Fuentes H., Rizvanov A., Mari B., Carraro G., Bellusci S. (2017). MicroRNA-142 is a multifaceted regulator in organogenesis, homeostasis, and disease. Dev. Dyn..

[116] Wang X.F., Shi Z.M., Wang X.R., Cao L., Wang Y.Y., Zhang J.X., Yin Y., Luo H., Kang C.S., Liu N. (2012). MiR-181d acts as a tumor suppressor in glioma by targeting K-ras and Bcl-2. J. Cancer Res. Clin. Oncol..

[117] Zhang G., Liu D., Long G., Shi L., Qiu H., Hu G., Hu G., Liu S. (2017). Downregulation of microRNA-181d had suppressive effect on pancreatic cancer development through inverse regulation of KNAIN2. Tumour Biol..

[118] Zhang H., Qi S., Zhang T., Wang A., Liu R., Guo J., Wang Y., Xu Y. (2015). miR-188-5p inhibits tumour growth and metastasis in prostate cancer by repressing LAPTM4B expression. Oncotarget.

[119] Wang L., Liu H. (2016). microRNA-188 is downregulated in oral squamous cell carcinoma and inhibits proliferation and invasion by targeting SIX1. Tumour Biol..

[120] Li N., Shi H., Zhang L., Li X., Gao L., Zhang G., Shi Y., Guo S. (2017). MiR-188 Inhibits Glioma Cell Proliferation and Cell Cycle Progression through Targeting ss-catenin. Oncol. Res..

[121] Peng Y., Shen X., Jiang H., Chen Z., Wu J., Zhu Y., Zhou Y., Li J. (2018). MiR-188-5p suppresses gastric cancer cell proliferation and invasion via targeting ZFP91. Oncol. Res..

[122] Yuva-Aydemir Y., Simkin A., Gascon E., Gao F.B. (2011). MicroRNA-9: Functional evolution of a conserved small regulatory RNA. RNA Biol..

[123] Sun H., Shao Y., Huang J., Sun S., Liu Y., Zhou P., Yang H. (2016). Prognostic value of microRNA-9 in cancers: A systematic review and meta-analysis. Oncotarget.

[124] Chang Y., Lin J., Tsung A. (2012). Manipulation of autophagy by MIR375 generates antitumor effects in liver cancer. Autophagy.

[125] Shi Z.C., Chu X.R., Wu Y.G., Wu J.H., Lu C.W., Lu R.X., Ding M.C., Mao N.F. (2015). MicroRNA-375 functions as a tumor suppressor in osteosarcoma by targeting PIK3CA. Tumour Biol..

[126] Cui F., Wang S., Lao I., Zhou C., Kong H., Bayaxi N., Li J., Chen Q., Zhu T., Zhu H. (2016). miR-375 inhibits the invasion and metastasis of colorectal cancer via targeting SP1 and regulating EMT-associated genes. Oncol. Rep..

[127] Xu L., Wen T., Liu Z., Xu F., Yang L., Liu J., Feng G., An G. (2016). MicroRNA-375 suppresses human colorectal cancer metastasis by targeting Frizzled 8. Oncotarget.

[128] Osako Y., Seki N., Kita Y., Yonemori K., Koshizuka K., Kurozumi A., Omoto I., Sasaki K., Uchikado Y., Kurahara H. (2016). Regulation of MMP13 by antitumor microRNA-375 markedly inhibits cancer cell migration and invasion in esophageal squamous cell carcinoma. Int. J. Oncol..

[129] Wei R., Yang Q., Han B., Li Y., Yao K., Yang X., Chen Z., Yang S., Zhou J., Li M. (2017). microRNA-375 inhibits colorectal cancer cells proliferation by downregulating JAK2/STAT3 and MAP3K8/ERK signaling pathways. Oncotarget.

[130] Wang Z., Hong Z., Gao F., Feng W. (2013). Upregulation of microRNA-375 is associated with poor prognosis in pediatric acute myeloid leukemia. Mol. Cell. Biochem..

[131] Costa-Pinheiro P., Ramalho-Carvalho J., Vieira F.Q., Torres-Ferreira J., Oliveira J., Goncalves C.S., Costa B.M., Henrique R., Jeronimo C. (2015). MicroRNA-375 plays a dual role in prostate carcinogenesis. Clin. Epigenet..

[132] Liu L., Liao J.Z., He X.X., Li P.Y. (2017). The role of autophagy in hepatocellular carcinoma: Friend or foe. Oncotarget.

[133] Qiu H.J., Lu X.H., Yang S.S., Weng C.Y., Zhang E.K., Chen F.C. (2016). MiR-769 promoted cell proliferation in human melanoma by suppressing GSK3B expression. Biomed. Pharmacother..

[134] Arora R., Shuda M., Guastafierro A., Feng H., Toptan T., Tolstov Y., Normolle D., Vollmer L.L., Vogt A., Domling A. (2012). Survivin is a therapeutic target in Merkel cell carcinoma. Sci. Transl. Med..

[135] Lasithiotaki I., Tsitoura E., Koutsopoulos A., Lagoudaki E., Koutoulaki C., Pitsidianakis G., Spandidos D.A., Siafakas N.M., Sourvinos G., Antoniou K.M. (2017). Aberrant expression of miR-21, miR-376c and miR-145 and their target host genes in Merkel cell polyomavirus-positive non-small cell lung cancer. Oncotarget.

[136] Hayes J., Peruzzi P.P., Lawler S. (2014). MicroRNAs in cancer: Biomarkers, functions and therapy. Trends Mol. Med..

[137] Lan H., Lu H., Wang X., Jin H. (2015). MicroRNAs as potential biomarkers in cancer: Opportunities and challenges. Biomed. Res. Int..

[138] Larrea E., Sole C., Manterola L., Goicoechea I., Armesto M., Arestin M., Caffarel M.M., Araujo A.M., Araiz M., Fernandez-Mercado M. (2016). New Concepts in Cancer Biomarkers: Circulating miRNAs in Liquid Biopsies. Int. J. Mol. Sci..

[139] Barger J.F., Rahman M.A., Jackson D., Acunzo M., Nana-Sinkam S.P. (2016). Extracellular miRNAs as biomarkers in cancer. Food Chem. Toxicol..

[140] Moretti F., D’Antona P., Finardi E., Barbetta M., Dominioni L., Poli A., Gini E., Noonan D.M., Imperatori A., Rotolo N. (2017). Systematic review and critique of circulating miRNAs as biomarkers of stage I-II non-small cell lung cancer. Oncotarget.

[141] Wang H., Peng R., Wang J., Qin Z., Xue L. (2018). Circulating microRNAs as potential cancers biomarkers: The advantage and disavantage. Clin. Epigenet..

[142] Huang X., Yuan T., Liang M., Du M., Xia S., Dittmar R., Wang D., See W., Costello B.A., Quevedo F. (2016). Exosomal miR-1290 and miR-375 as prognostic markers in castration-resistant prostate cancer. Oncol. Rep..

[143] Pfeffer S.R., Grossmann K.F., Cassidy P.B., Yang C.H., Fan M., Kopelovich L., Leachman S.A., Pfeffer L.M. (2015). Detection of Exosomal miRNAs in the Plasma of Melanoma Patients. J. Clin. Med..

[144] Imamura T., Komatsu S., Ichikawa D., Miyamae M., Okajima W., Ohashi T., Kiuchi J., Nishibeppu K., Kosuga T., Konishi H. (2017). Low plasma levels of miR-101 are associated with tumor progression in gastric cancer. Oncotarget.

[145] Zhao Q., Deng S., Wang G., Liu C., Meng L., Qiao S., Shen L., Zhang Y., Lu J., Li W. (2016). A direct quantification method for measuring plasma MicroRNAs identified potential biomarkers for detecting metastatic breast cancer. Oncotarget.

[146] Foj L., Ferrer F., Serra M., Arevalo A., Gavagnach M., Gimenez N., Filella X. (2017). Exosomal and Non-Exosomal Urinary miRNAs in Prostate Cancer Detection and Prognosis. Prostate.

[147] Wang J., Yan F., Zhao Q., Zhan F., Wang R., Wang L., Zhang Y., Huang X. (2017). Circulating exosomal miR-125a-3p as a novel biomarker for early-stage colon cancer. Sci. Rep..

[148] Butz H., Nofech-Mozes R., Ding Q., Khella H.W.Z., Szabo P.M., Jewett M., Finelli A., Lee J., Ordon M., Stewart R. (2016). Exosomal MicroRNAs Are Diagnostic Biomarkers and Can Mediate Cell-Cell Communication in Renal Cell Carcinoma. Eur. Urol. Focus.

[149] Jiang Q., Lagos-Quintana M., Liu D., Shi Y., Helker C., Herzog W., le Noble F. (2013). miR-30a regulates endothelial tip cell formation and arteriolar branching. Hypertension.

[150] Alsaleh G., Francois A., Philippe L., Gong Y.Z., Bahram S., Cetin S., Pfeffer S., Gottenberg J.E., Wachsmann D., Georgel P. (2014). MiR-30a-3p negatively regulates BAFF synthesis in systemic sclerosis and rheumatoid arthritis fibroblasts. PLoS ONE.

[151] Muther C., Jobeili L., Garion M., Heraud S., Thepot A., Damour O., Lamartine J. (2017). An expression screen for aged-dependent microRNAs identifies miR-30a as a key regulator of aging features in human epidermis. Aging.

[152] Kapsimali M., Kloosterman W.P., de Bruijn E., Rosa F., Plasterk R.H., Wilson S.W. (2007). MicroRNAs show a wide diversity of expression profiles in the developing and mature central nervous system. Genome Biol..

[153] Lu T.X., Lim E.J., Wen T., Plassard A.J., Hogan S.P., Martin L.J., Aronow B.J., Rothenberg M.E. (2012). MiR-375 is downregulated in epithelial cells after IL-13 stimulation and regulates an IL-13-induced epithelial transcriptome. Mucosal Immunol..

[154] Eliasson L. (2017). The small RNA miR-375—A pancreatic islet abundant miRNA with multiple roles in endocrine beta cell function. Mol. Cell. Endocrinol..

[155] Branch S., Maloney K., Purcell S.M. (2018). Spontaneous regression of Merkel cell carcinoma. Cutis.

[156] Ahmadi Moghaddam P., Cornejo K.M., Hutchinson L., Tomaszewicz K., Dresser K., Deng A., O’Donnell P. (2016). Complete Spontaneous Regression of Merkel Cell Carcinoma After Biopsy: A Case Report and Review of the Literature. Am. J. Dermatopathol..

[157] Cirillo F. (2015). Spontaneous Regression of Primitive Merkel Cell Carcinoma. Rare Tumors.

[158] Walsh N.M. (2016). Complete spontaneous regression of Merkel cell carcinoma (1986–2016): A 30 year perspective. J. Cutan. Pathol..

[159] Mantripragada K., Birnbaum A. (2015). Response to Anti-PD-1 Therapy in Metastatic Merkel Cell Carcinoma Metastatic to the Heart and Pancreas. Cureus.

[160] Nghiem P.T., Bhatia S., Lipson E.J., Kudchadkar R.R., Miller N.J., Annamalai L., Berry S., Chartash E.K., Daud A., Fling S.P. (2016). PD-1 Blockade with Pembrolizumab in Advanced Merkel-Cell Carcinoma. N. Engl. J. Med..

[161] Kaufman H.L., Russell J., Hamid O., Bhatia S., Terheyden P., D’Angelo S.P., Shih K.C., Lebbe C., Linette G.P., Milella M. (2016). Avelumab in patients with chemotherapy-refractory metastatic Merkel cell carcinoma: A multicentre, single-group, open-label, phase 2 trial. Lancet Oncol..

[162] Walocko F.M., Scheier B.Y., Harms P.W., Fecher L.A., Lao C.D. (2016). Metastatic Merkel cell carcinoma response to nivolumab. J. Immunother. Cancer.

[163] Thiem A., Kneitz H., Schummer P., Herz S., Schrama D., Houben R., Goebeler M., Schilling B., Gesierich A. (2017). Coincident Metastatic Melanoma and Merkel Cell Carcinoma with Complete Remission on Treatment with Pembrolizumab. Acta Derm. Venereol..

[164] Zhao C., Tella S.H., Del Rivero J., Kommalapati A., Ebenuwa I., Gulley J., Strauss J., Brownell I. (2018). Anti-PD-L1 Treatment Induced Central Diabetes Insipidus. J. Clin. Endocrinol. Metab..

[165] Kaufman H.L., Russell J.S., Hamid O., Bhatia S., Terheyden P., D’Angelo S.P., Shih K.C., Lebbe C., Milella M., Brownell I. (2018). Updated efficacy of avelumab in patients with previously treated metastatic Merkel cell carcinoma after >/=1 year of follow-up: JAVELIN Merkel 200, a phase 2 clinical trial. J. Immunother. Cancer.

[166] Eshghi N., Lundeen T.F., MacKinnon L., Avery R., Kuo P.H. (2018). 18F-FDG PET/CT for Monitoring Response of Merkel Cell Carcinoma to the Novel Programmed Cell Death Ligand 1 Inhibitor Avelumab. Clin. Nucl. Med..

[167] Topalian S.L., Bhatia S., Hollebecque A., Awada A., de Boer J.P., Kudchadkar R.R., Goncalves A., Delord J.-P., Martens U.M., Picazo J.M.L. (2017). Non-comparative, open-label, multiple cohort, phase 1/2 study to evaluate nivolumab (NIVO) in patients with virus-associated tumors (CheckMate 358): Efficacy and safety in Merkel cell carcinoma (MCC). Cancer Res..

[168] Winkler J.K., Dimitrakopoulou-Strauss A., Sachpekidis C., Enk A., Hassel J.C. (2017). Ipilimumab has efficacy in metastatic Merkel cell carcinoma: A case series of five patients. Eur. Acad. Dermatol. Venereol..

[169] Williams B.A., Hardee M.E., Hutchins L.F., Shalin S., Gao L. (2015). A case of Merkel cell carcinoma treatment with anti-CTLA-4 antibody (Ipilimumab). J. Clin. Case Rep..

[170] Shah M.Y., Ferrajoli A., Sood A.K., Lopez-Berestein G., Calin G.A. (2016). microRNA Therapeutics in Cancer—An Emerging Concept. EBioMedicine.

[171] Rupaimoole R., Slack F.J. (2017). MicroRNA therapeutics: Towards a new era for the management of cancer and other diseases. Nat. Rev. Drug Discov..

[172] Chakraborty C., Sharma A.R., Sharma G., Doss C.G.P., Lee S.S. (2017). Therapeutic miRNA and siRNA: Moving from Bench to Clinic as Next Generation Medicine. Mol. Ther. Nucleic Acids.

[173] Verma M., Lam T.K., Hebert E., Divi R.L. (2015). Extracellular vesicles: Potential applications in cancer diagnosis, prognosis, and epidemiology. BMC Clin. Pathol..

[174] Maia J., Caja S., Strano Moraes M.C., Couto N., Costa-Silva B. (2018). Exosome-Based Cell-Cell Communication in the Tumor Microenvironment. Front. Cell Dev. Biol..

[175] Graner M.W., Schnell S., Olin M.R. (2018). Tumor-derived exosomes, microRNAs, and cancer immune suppression. Semin. Immunopathol..

